# Endothelin-1 contributes to the development of virus-induced demyelinating disease

**DOI:** 10.1186/s12974-020-01986-z

**Published:** 2020-10-17

**Authors:** Young-Hee Jin, Bongsu Kang, Hyun S. Kang, Chang-Sung Koh, Byung S. Kim

**Affiliations:** 1 Department of Microbiology-Immunology, Northwestern University Feinberg Medical School, 303 East Chicago Avenue, Chicago, IL 60611 USA; 2 KM Application Center, Korea Institute of Oriental Medicine, Daegu, Republic of Korea; 3 Center for Convergent Research of Emerging Virus Infection, Korea Research Institute of Chemical Technology, Daejeon, Republic of Korea; 4 Department of Biomedical Laboratory Sciences, Graduate School of Medicine, Shinshu University, Matsumoto, Nagano, 390-8621 Japan

**Keywords:** CNS, EAE, TMEV, Endothelin-1, Demyelination

## Abstract

**Background:**

Experimental autoimmune encephalitis (EAE) and virally induced demyelinating disease are two major experimental model systems used to study human multiple sclerosis. Although endothelin-1 level elevation was previously observed in the CNS of mice with EAE and viral demyelinating disease, the potential role of endothelin-1 in the development of these demyelinating diseases is unknown.

**Methods and results:**

In this study, the involvement of endothelin-1 in the development and progression of demyelinating diseases was investigated using these two experimental models. Administration of endothelin-1 significantly promoted the progression of both experimental diseases accompanied with elevated inflammatory T cell responses. In contrast, administration of specific endothelin-1 inhibitors (BQ610 and BQ788) significantly inhibited progression of these diseases accompanied with reduced T cell responses to the respective antigens.

**Conclusions:**

These results strongly suggest that the level of endothelin-1 plays an important role in the pathogenesis of immune-mediated CNS demyelinating diseases by promoting immune responses.

## Introduction

Theiler’s murine encephalomyelitis virus (TMEV) causes a chronic progressive demyelinating disease in susceptible mice [1, 2]. Development of this virally induced demyelinating disease appears to be immune-mediated and primarily involves CD4^+^ T cells [3, 4]. Infiltration of proinflammatory T cells such as Th17 and Tc17 appears to be associated with tissue destruction and demyelination [5, 6], similar to multiple sclerosis (MS) [7–9]. In addition, both T cell and antibody responses to self-antigens are induced in susceptible mice after chronic TMEV infection [10–13]. Therefore, this viral demyelinating disease model in mice has been investigated as a relevant animal model for MS. Infection of cells with TMEV activates production of various cytokines via TLR- and melanoma differentiation-associated gene 5 (MDA5)-dependent pathways [14–16]. High levels of IL-1 or type I IFNs, which are downstream products of TLR or MDA5 activation, play a pathogenic role [17, 18]. Similarly, excessive levels of IL-6 exert a potent pathogenic effect on the development of TMEV-induced demyelinating disease by promoting Th17 responses [5]. Furthermore, TLR-mediated signals induce PGE_2_ elevation via the NLRP3 pathway to affect the pathogenesis of TMEV-induced demyelinating disease [19].

Moreover, TMEV infection activates production of various chemokines in different glial cell types [20, 21]. These proinflammatory chemokines consequently contribute to cellular infiltration into the CNS and further activation of the infiltrating inflammatory immune cells [22–24], eventually leading to the development and progression of virus-induced demyelinating disease. However, very little is known about the role of vascular components such as VEGF in the pathogenesis of TMEV-induced demyelinating disease as VEGF also appears to be associated with pathological changes in MS and its animal models [25–27]. Similarly, endothelin-1 (ET-1) levels appear to affect the pathogenesis of both MS and its animal model disease [28–30]. Therefore, it is valuable to investigate whether ET-1 contributes as a common mechanism in the development of TMEV-induced demyelinating disease.

Several reports indicate that TLR2 and TLR4 agonists induce ET-1 production in human DCs [31]. In addition, TLR3 activation also induced ET-1 production [32, 33]. Since TMEV infection is known to activate various cellular proteins via TLR2 and TLR3 [14, 15, 34], we predict that TMEV infection induces ET-1 via TLR signaling, which may affect the development of TMEV-induced demyelinating disease. ET-1 is produced in many different cell types such as endothelial cells, neurons, and macrophages [35]. ET-1 is associated with the activation of transcription factors such as NF-κB and expression of proinflammatory cytokines including TNF-α, IL-1, and IL-6 [36]. In addition, ET-1 enhances VCAM-1 expression on vascular endothelial cells [37]. In addition, ET-1 induces COX-2 expression and PGE2 production [38]. ET-1 is known to function by engaging with its G-protein-coupled receptors, mainly ETA and ETB [39]. Therefore, it is conceivable that ET-1 induced via TLR activation following TMEV infection may also participate in the pathogenesis of demyelinating disease.

To investigate the aforementioned possibility, we examined the potential role of ET-1 in the development of TMEV-induced demyelinating disease. In this study, we established that TMEV-infected susceptible SJL mice displayed an elevated level of ET-1 compared to uninfected mice. In addition, ET-1 administration significantly promoted progression of TMEV-induced demyelinating disease accompanied with increased cellular infiltration of the CNS and elevated inflammatory T cell responses. These results suggest that the ET-1 induced following TMEV infection contributes to development of viral demyelinating disease. To further investigate this possibility, we administered specific inhibitors (BQ610 and BQ788) for endothelin-1 receptors (ETA and ETB, respectively). These ET-1 receptor inhibitors significantly hindered disease progression accompanied with reduced cellular infiltration of the CNS and T cell responses to the viral antigens. Therefore, the results of this study strongly suggest that endothelin-1 levels significantly contribute to the pathogenesis of immune-mediated CNS demyelinating diseases by promoting inflammatory immune responses following the neurotrophic TMEV infection.

## Materials and methods

### Animals

Female SJL/J (SJL) mice (4–6 weeks old) were purchased from Charles River Laboratories (Charles River, MA) through the National Cancer Institute (Frederick, MD). All mice were housed at the Center for Comparative Medicine Facility, Northwestern University. All animal studies used protocols approved by the Institutional Animal Care and Use Committee.

### Viruses

BeAn 8386 strain of TMEV was propagated in BHK-21 cells in Dulbecco’s modified Eagle’s medium supplemented with 7.5% donor calf serum. For some experiments, the virus was purified by centrifugation on sucrose gradients, as previously described [40].

### Chemicals

The peptide representing the mature form of endothelin-1 was purchased from Sigma-Aldrich (St. Louis, MO). Endothelin-1 inhibitors, namely, BQ610 for endothelin receptor-A (ETA) and BQ788 for endothelin receptor-B (ETB), were also obtained from Sigma-Aldrich.

### Synthetic peptides

All synthetic peptides were purchased from Genmed Synthesis (San Francisco, CA). Stock peptides of the previously defined TMEV-specific CD4^+^ (VP2_203-220_ and VP4_25-38_) and CD8^+^ T cell epitopes (VP2_121-130_ and VP3_110-120_) for SJL mice were prepared in a solution of 8% dimethylsulfoxide (DMSO) in phosphate-buffered saline (PBS).

### TMEV infection

Viral titer was determined through a plaque assay on BHK cells; this was used to determine the multiplicity of infection. For animal studies, 1 × 10^6^ PFU of TMEV in 30 μl DMEM was injected into the right cerebral hemisphere of female SJL/J mice anesthetized with isoflurane. Clinical symptoms of disease were assessed weekly based on the following grading scale [41]: grade 0, no clinical signs; grade 1, mild waddling gait; grade 2, moderate waddling gait and hind-limb paresis; grade 3, severe hind-limb paralysis; grade 4, severe hind-limb paralysis and loss of righting reflex; and grade 5, death.

### EAE induction

SJL/J female mice were subcutaneously immunized twice (days 0 and 7) with PLP_139-151_ (40 μg/mouse) in incomplete adjuvant containing H37RA (100 μg/mouse) according to the injection schedule previously reported [42].

### ET-1 and ET receptor antagonist treatment

One hundred picomoles of 30 μl ET-1 containing 1 × 10^6^ PFU TMEV was initially injected intracerebrally. Thirty days after the first injection, 100 pmol of 30 μl ET-1 without TMEV was injected either i.c. or i.v. For experiments involving ET receptor antagonists, PBS (cont), BQ610, or BQ788 (1 mg/kg) was injected i.v. into the SJL mice multiple times at days 0, 5, 10, 15, 20, 30, and 46 post-viral infection.

### CNS mononuclear cell preparation

The brains and spinal cords were removed from the mice after perfusion with Hank’s balanced salt solution through the left ventricle. Tissues were forced through a steel mesh to prepare single-cell suspensions and were incubated at 37 °C for 45 min in 250 μg/ml collagenase type 4 (Worthington Biochemical Corp., Lakewood, NJ, USA). A continuous 100% Percoll gradient (Pharmacia, Piscataway, NJ, USA) was created by centrifuging at 27,000×*g* for 30 min to enrich CNS-infiltrating mononuclear cells as previously described [43].

### T cell proliferation assay

Spleen cells (1 × 10^6^ cells/well) were stimulated with the indicated stimuli in 96 well flat-bottom microtiter plates in RPMI 1640 containing 0.5% syngeneic mouse serum and 5 × 10^− 5^ M 2-mercaptoethanol. After incubation with the antigens for 72 h, cultures were pulsed with 1.0 μCi of [^3^H] TdR and harvested 18 h later. Measurements of the [^3^H] TdR uptake by the cells was performed, and these were expressed as counts per minute (Δcpm) +/− SEM) after subtracting the background count with PBS. Triplicate cultures were stimulated with either PLP_139-151_ (10 μg) for EAE mice, UV-inactivated TMEV (1, 3 μg), or TMEV T cell epitope peptides (at 1, 10 μM of VP1_233-250_, VP2_74-86_, VP3_24-37_) for TMEV-infected mice. As an unrelated peptide control, hen egg lysozyme (HEL_47-61_) was used.

### Histopathological staining

At 30 and 60 days post-TMEV infection, mice were perfused with 50 ml of PBS via intracardiac puncture. The brain and spinal cords from ET1-treated or untreated SJL mice were dissected, and these were fixed in 4% formalin in PBS for 4 days, transferred into 30% sucrose/PBS solution and incubated for 24 h, and embedded in paraffin. Paraffin-processed brain and spinal cord samples were sectioned with a thickness of 6 μm, and two sets of adjacent sections from each animal were deparaffinized, rehydrated, and separately evaluated using Luxol fast blue (LFB) staining for axonal demyelination, which were then counterstained with hematoxylin and eosin (H&E) to detect inflammatory infiltrates and Bielschowsky silver staining for observing axon damage and loss.

### RT-PCR and real-time PCR

Total RNA was extracted from the lysates of the brain/spinal cord cells using TRIzol (Invitrogen, Carlsbad, CA, USA) according to the manufacturer’s instructions. First-strand cDNA was synthesized using MMLV reverse transcriptase and oligo (dT)_18_ from 1–4 μg total RNA depending on the frequencies of the transcripts. MJ Research, Inc. (Watertown, MA, USA) thermal cycler was used for PCR. Primers were obtained from Integrated DNA Technologies (Coralville, IA, USA). Sense and antisense primer sequences used are as follows: ET-1 (5’-AGAGTGTGTCTACTTCTGCC-3’ and 5’-GCGTTATGTGACCC-ACAAC-3’); CCL2 (5’-AGCAGGTGTCCCAAAGAAGCTGTA-3’ and 5’-AGAAGTGCT-TGAGGTGGTTGTGGA-3’); CXCL10 (5’ -AAGTGCTGCCGTCATTTTCT-3’ and 5’ -GTGGCAATGATCTCAACACG-3’); CXCL1 (5’-GCTGGGATTCACCTCAAGAA-3’ and 5’-TGGGGACACCT-TTTAGCATC-3’); IFN-γ (5’-ACTGGCAAAAGGATGGTGAC-3’ and 5’-TGAGCTCATTGAATGCTTGG-3’); IL-17A (5’-CTCCAGAAGGCCCTCA-GACTAC-3’ and 5’-AGC-TTTCCCTCCGCATTGACACAG-3’); IL-10; (5’-GCCAAGCCTTATCGGAAATG-ATCC-3’ and 5’-AGACACCTTGGTCTTGGAGCTT-3’); IL-12 (5’-CAGAAGCTAACC-ATCTCCTGGTTTG-3′ and 5′-TCCGGAGTAATTTGGTG CTTCACAC-3′); CD4 (5’-TGTGCCGAGCCATCTCTCTTAGG-3’ and 5’-GCACTGAGAGTGTCATGCC-GAAC-3’); CD8 (5’-TCTGTCGTG CCAGTCCTTC-3’ and 5’-CCTTCCTGTCTGACTAGC GG-3’); GAPDH (5’-AACTTTGGC-ATTGTGGAAGG-3’ and 5’-ACACATTGGGGGTAGGAACA-3’); and TMEV genome (5’-CCCAGTCCTCAGGAAATGAAGG-3’ and 5’-TCCAAAAGG-AGAGGTGCCATAG-3’). For quantitative analysis of gene expression, real-time PCR was performed using the iCycler SYBR Green I master mix and an iCycler Real-Time PCR System (Bio-Rad, Hercules, CA, USA). GAPDH expression was used for normalization of gene expression levels. The expression level represents the fold-increase compared to the lowest values in the group. Real-time PCR reactions were performed in triplicate.

### ELISA assay for detection of IFN-γ protein

ELISA was used to assess cytokine levels produced by splenocytes in response to the PLP and TMEV epitopes. Briefly, splenocytes (2–3 × 10^6^ cells/well) from TMEV-infected SJL/J mice were stimulated with various concentrations of either peptides or UV-TMEV for 72 h. Cell-free supernatants were examined for the presence of IFN-γ through cytokine capture ELISA using the OptEIA kit (BD Pharmingen, San Diego, CA).

### Statistical analyses

Significant differences (two-tailed *p* value) between the experimental groups with various treatments and the control group were analyzed through an unpaired Student’s *t* test (unless otherwise indicated) using the InStat Program (GraphPAD Software, San Diego, CA, USA). Multiple group comparisons were done using a one-way analysis of variance with Tukey-Kramer post hoc analysis. Values of *p* < 0.05 were considered significant.

## Results

### Elevated ET-1 mRNA expression in SJL mice after TMEV infection

**Fig. 1:**
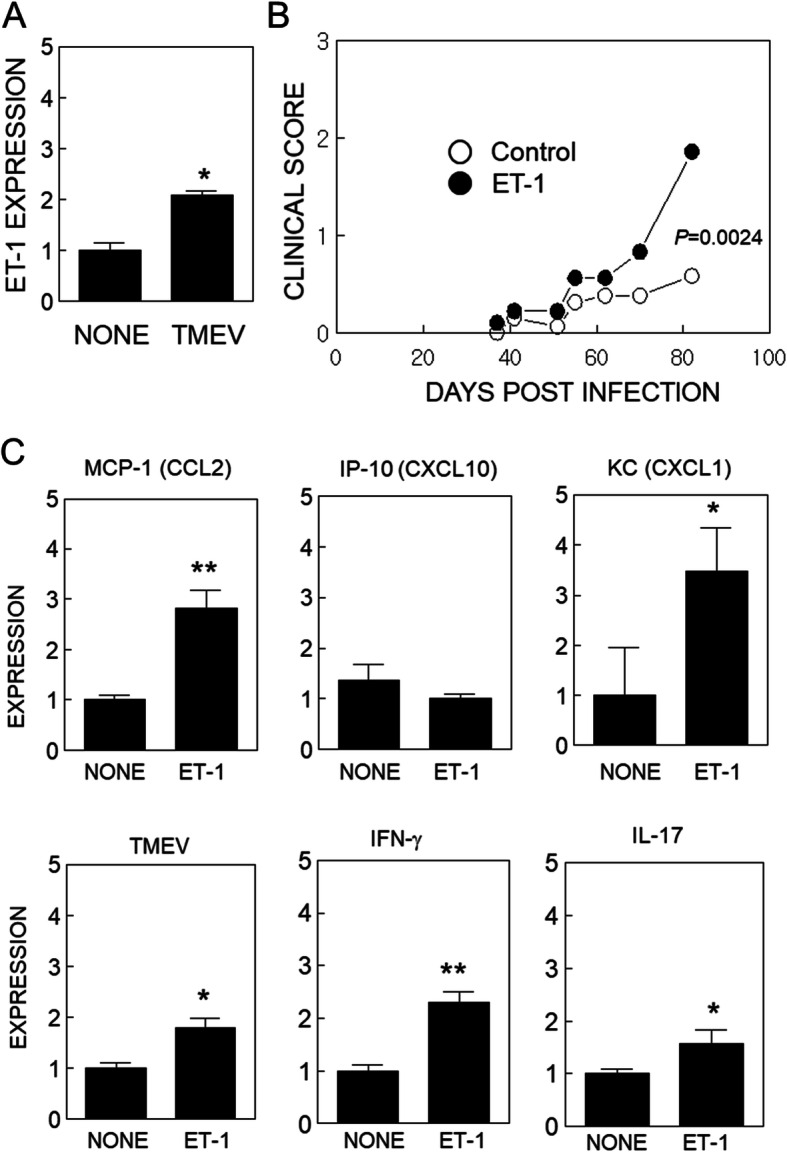
Effect of endothelin-1 on the development of Theiler’s murine encephalomyelitis virus (TMEV)-induced demyelinating disease. **a** Levels of ET-1 mRNA in the CNS of naïve mice or mice infected with TMEV at 8 dpi. **b** SJL/J mice (*n* = 10/group) were intracerebrally infected with a suboptimal dose (1 × 10^5^ PFU) of TMEV and were treated with either PBS or ET-1 (20 μg/kg) at days − 3, 0, 5, 10, 15, 20, and 46. Disease development was evaluated based on the 5-point scale described in the “Materials and methods” section. The difference between the control and ET-1 groups was significant (*p* < 0.0024) based on the paired two-tailed *t* test between 37 and 85 dpi. **c** Relative expression of MCP-1 (CCL2), IP-10 (CXCL-10), KC (CXCL1), TMEV, IFN-γ, and IL-17A mRNA. Real-time PCR was used to assess expression levels. Data are presented as mean ± SEM of three independent experiments. *p < 0.05 and **p < 0.001

TLR activation is associated with ET-1 induction, which has been associated with the pathogenesis of MS and shown to contribute to experimental models for the disease. Therefore, we first tested the possibility that SJL mice infected with TMEV exhibit elevated ET-1 levels in the CNS (Fig. 1a). When the ET-1 mRNA levels in the CNS of SJL mice were compared between uninfected and TMEV-infected mice at 8 days post-infection by real time-PCR, ET-1 mRNA was significantly elevated in TMEV-infected mice compared to uninfected mice. This result is consistent with previous observations that TLR activation following viral infection may elevate ET-1 production [32, 33].

### Exacerbation of TMEV-IDD in SJL mice following administration of ET-1

To further investigate the possibility that elevated ET-1 production following viral infection affects the pathogenesis of TMEV-induced demyelinating disease, we administered the ET-1 peptide during the viral infection (Fig. 1b). SJL mice (*n* = 10/group) were infected with a suboptimal dose (1 × 10^5^ PFU) of TMEV. The experimental group was treated with ET-1 (20 μg/kg) and the control group was treated with PBS at days − 3, 0, 5, 10, 15, 20, and 46 with respect to viral infection. The development of clinical symptoms was significantly accelerated (*p* < 0.0024) in mice treated with ET-1 compared to the control mice. ET-1 treated mice also showed elevated levels of chemokines (CCL2 and CXCL1) affecting cellular infiltration at 8 dpi (Fig. 1c upper panel). However, CXCL10 was not elevated in mice treated with ET-1. In addition, the levels of viral RNA and cytokine messages (IFN-γ and IL-17A) were significantly increased (Fig. 1c, lower panel). Therefore, ET-1 elevation may affect the upregulation of select chemokines, cytokines, and viral RNA in conjunction with TMEV infection.

### Administration of ET-1 increase the infiltration of various inflammatory cells

**Fig. 2:**
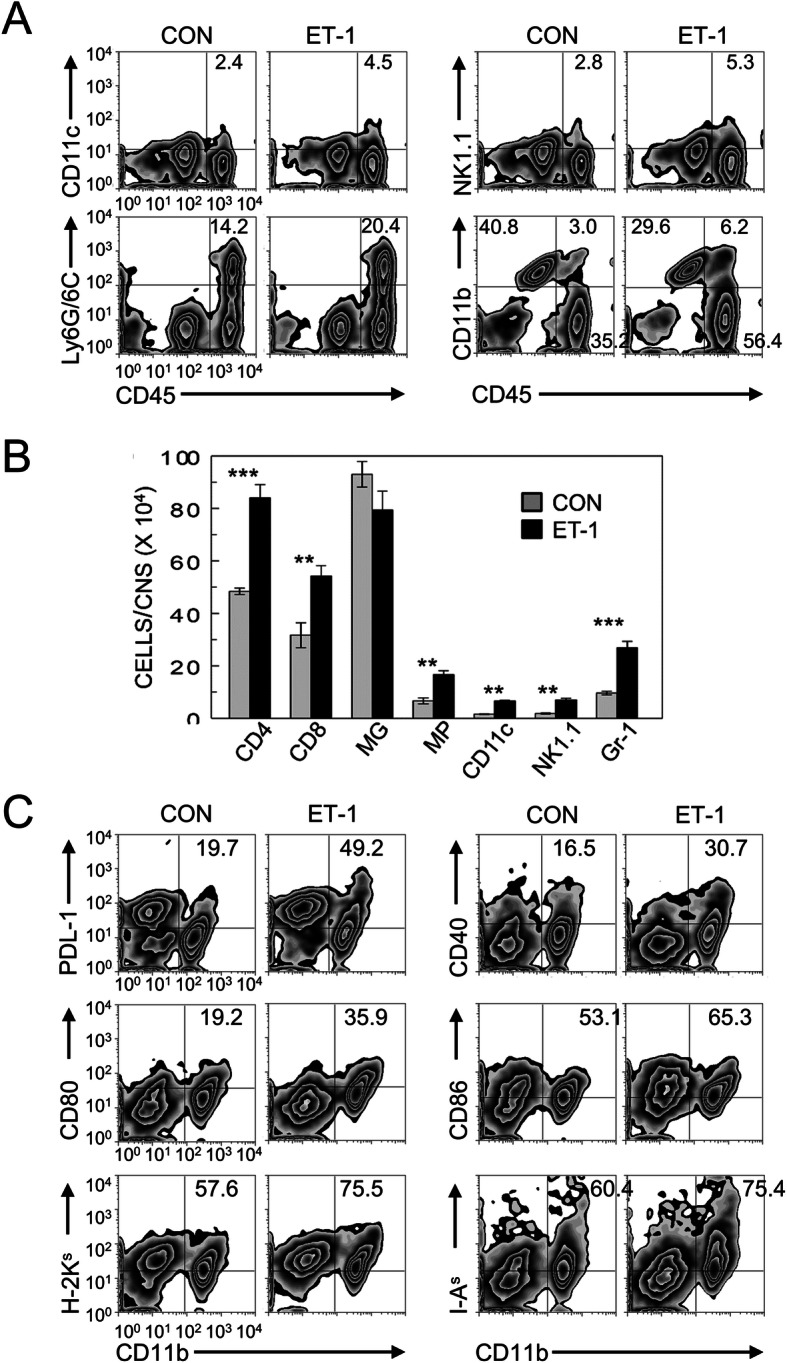
Effects of endothelin administration on the expression of inhibitory and co-stimulatory molecules on monocytes (CD11b^+^) in the CNS of infected mice. **a** Flow cytometric analyses of CNS-infiltrating cells at 8 dpi. A representative set of plots is shown. CD11c^+^ cells between control vs. ET-1 groups of 3–5 experiments, 2.23 ± 0.18 vs. 4.4 ± 0.11; NK1.1^+^ cells, 2.6 ± 0.25 vs. 5.0 ± 0.37; Ly6G/6C^+^ cells, 13.49 ± 1.00 vs. 19.2 ± 1.69; CD11b^+^CD45^int^ (microglia), 37.20 ± 1.93 vs. 26.46 ± 2.39; CD11b^+^CD45^hi^ (macrophage), 2.65 ± 0.45 vs. 5.55 ± 0.49, respectively. Mean ± SD is shown for each cell type. **b** Cell numbers of the above cell types in the CNS at 8 dpi, as determined by flow cytometry, are shown in the histogram. Differences in the cell numbers of the cell types except the microglia are significant. Data are presented as mean ± SEM of three independent experiments. **p < 0.001 and ***p < 0.0001. **c** Expression levels of inhibitory (PDL-1) and co-stimulatory molecules (CD40, CD80, CD86, and MHC) on the CNS CD11b^+^ cells in TMEV-infected mice treated with either PBS or ET-1 at 8 dpi (*n* = 3). The number indicated the % of marker expressed cells of total CD11b^+^ cell. Data are representative of three independent experiments

Because ET-1 can promote cellular migration into the CNS during inflammatory response [44], the levels of cell types that infiltrated the CNS after TMEV infection were compared between the groups treated with ET-1 and PBS (Fig. 2). Proportions of DCs (CD11c^+^), NK cells (NK1.1^+^), macrophages (CD45^+^CD11b^high^), and granulocytes (Ly6G/6C^+^, Gr-1^+^) that infiltrated the CNS at 8 dpi were first compared (Fig. 2a). Interestingly, ET-1 treated mice showed significantly higher proportions (~ 2-fold) and numbers of these cell types in the CNS compared to the control group (Fig. 2b). CD11b^+^ cells from ET-1-treated mice expressed elevated levels of co-stimulatory (CD40, CD80, and CD86) and MHC molecules (H-2K^s^ and I-A^s^), as well as PDL-1, compared with those from untreated control mice (Fig. 2c and Additional file 1: Supplementary Figure 1).

### Elevation of Th1 responses to viral antigens in TMEV-infected mice receiving ET-1

**Fig. 3:**
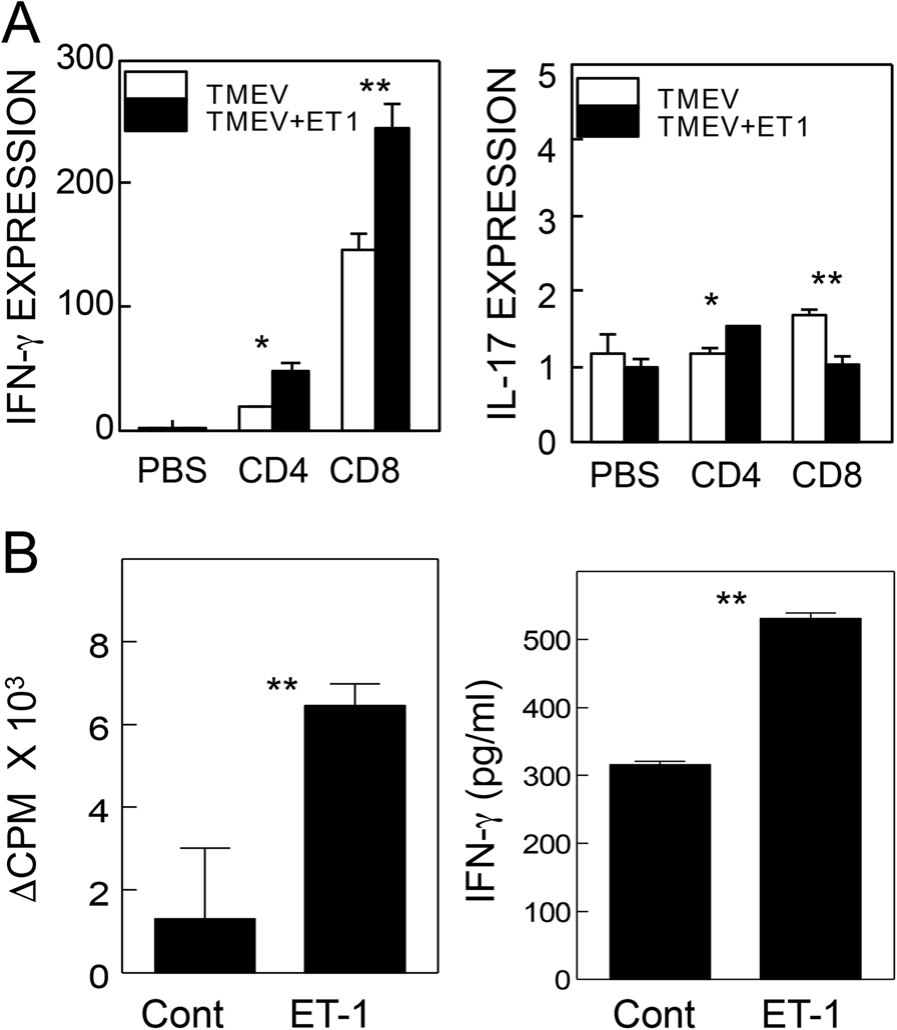
Cytokine levels in the CNS and splenocytes of TMEV-infected ET-1 treated mice. **a** Levels of cytokines produced by T cells in the CNS of SJL mice at 8 dpi were analyzed by real-time PCR following in vitro re-stimulation with CD4 T cell epitope VP2_203-220_ and VP4_25-38_ (CD4) and CD8 T cell epitopes VP2_121-130_ and VP3_110-120_ (CD8) for 6 h. Levels of IFN-γ and IL-17A T cell cytokine levels are shown. **b** T cell proliferation and IFN-γ produced after stimulation of CD4^+^ T cells with UV-TMEV for 4 days were measured using thymidine uptake and ELISA, respectively. Proliferative response of splenic T cells from control and ET-1-treated mice (*n* = 3) in response to UV-TMEV (5 μg) at 8 days after TMEV infection is shown. Levels of IFN-γ produced by T cells in the CNS following in vitro re-stimulation with either PBS or TMEV antigens are shown. Data are presented as mean ± SEM of three independent experiments. *p < 0.05 and **p < 0.001

To determine whether Th subtype responses are altered in mice treated with ET-1 compared to those in the control mice, mRNA levels of IFN-γ and IL-17A in the CNS of virus-infected mice at 8 dpi were determined using quantitative PCR after in vitro re-stimulation with peptides containing CD4 and CD8 T cell epitopes (Fig. 3a). IFN-γ mRNA levels in both CD4^+^ and CD8^+^ T cells were significantly higher in mice treated with ET-1 compared to the control mice. However, IL-17 mRNA levels were higher in CD4^+^ T cells but were lower in CD8^+^ T cells in ET-1 treated mice. The proliferation and IFN-γ secretion of splenocytes from ET-1-treated mice were significantly higher in response to UV-TMEV compared to those in untreated control mice (Fig. 3b).

### Histological examinations of TMEV-infected SJL mice treated with ET-1

**Fig. 4:**
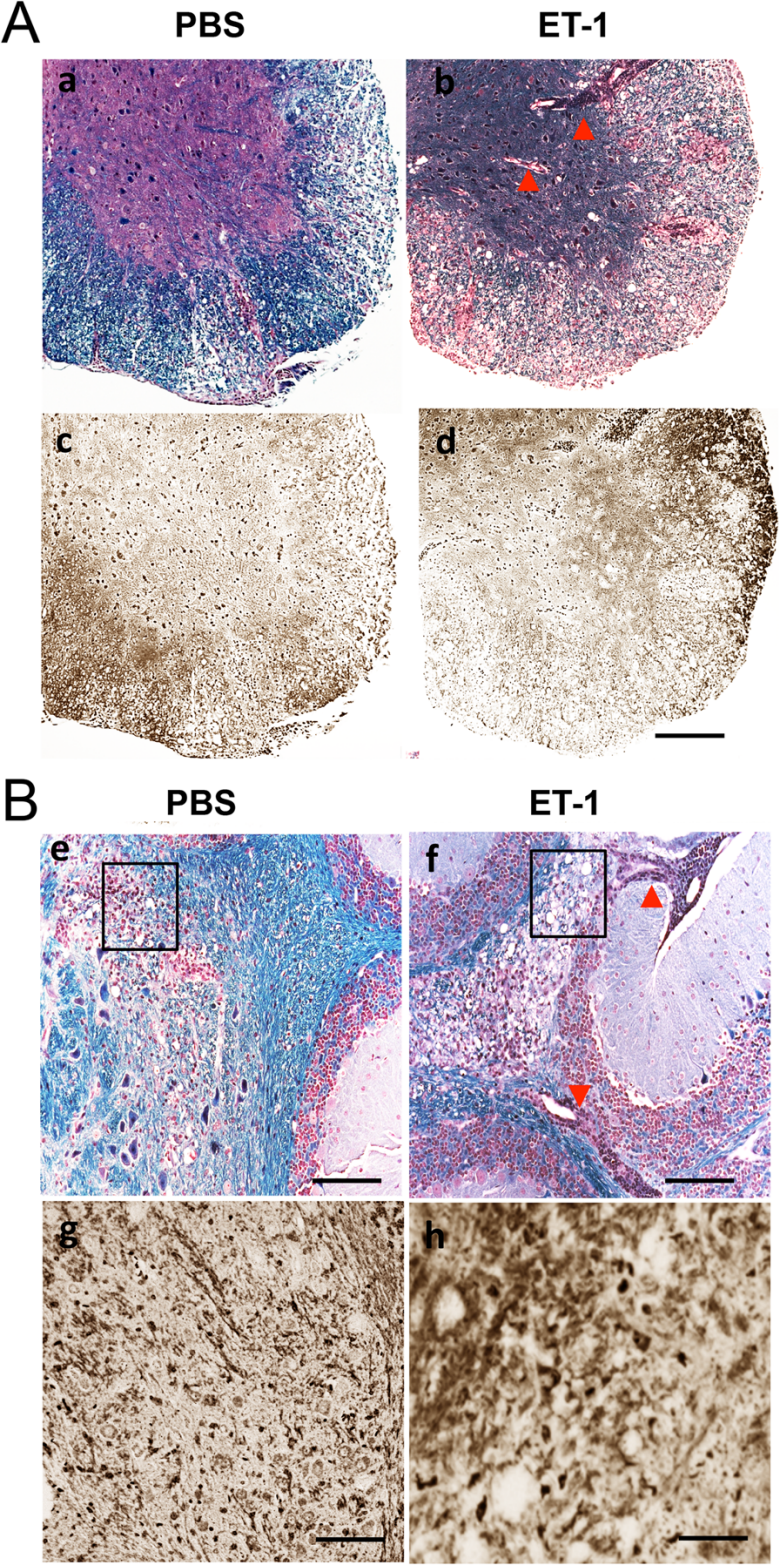
Histopathological changes in the CNS of ET-1 treated and untreated SJL mice on 30 days post-TMEV infection. **A** Spinal cord sections of PBS (a) and ET-1 (b) treated mice were stained with LFB and counterstained through H&E staining or through Bielschowsky silver staining (c, d), respectively. Scale bars, a–d = 200μm. **B** Cerebellum sections of PBS (e) and ET-1 (f) treated mice were stained with LFB/H&E or Bielschowsky silver staining of the adjacent sections (g, h), respectively. g and h showed the high magnification of the boxes in e and f. Scale bars, e–f = 100μm; g–h = 50μm. Data are representative of three independent experiments with blinded sample labels

To correlate the development of clinical signs with demyelination, the spinal cords (Fig. 4a) and brains (Fig. 4b) of mice infected with TMEV at 30 dpi were histologically assessed. Representative sections showed that there are more infiltrating lymphocytes/macrophages (indicated by arrowhead) that accumulated in the spinal cord (Fig. 4 Ab) and cerebellum (Fig. 4 Bf) of the ET-1-treated SJL mice infected with TMEV compared to those (Fig. 4 Aa and Be) in the control SJL mice. Infiltration mainly occurred in the white matter of the control SJL mice. However, in ET-1-treated SJL mice, infiltrated lymphocyte/macrophages invaded the gray matter of the spinal cord and cerebellum. Bielschowsky silver staining of the adjacent sections (Fig. 4 Ac, Ad, Bg, and Bh) showed axon damage in the demyelination region in both groups and also in the clustered lymphocyte/macrophages infiltrated area in the ET-1 treated mice brain.

### Inhibition of the development of TMEV-induced demyelinating disease after administration of ET-1 receptor inhibitors

**Fig. 5:**
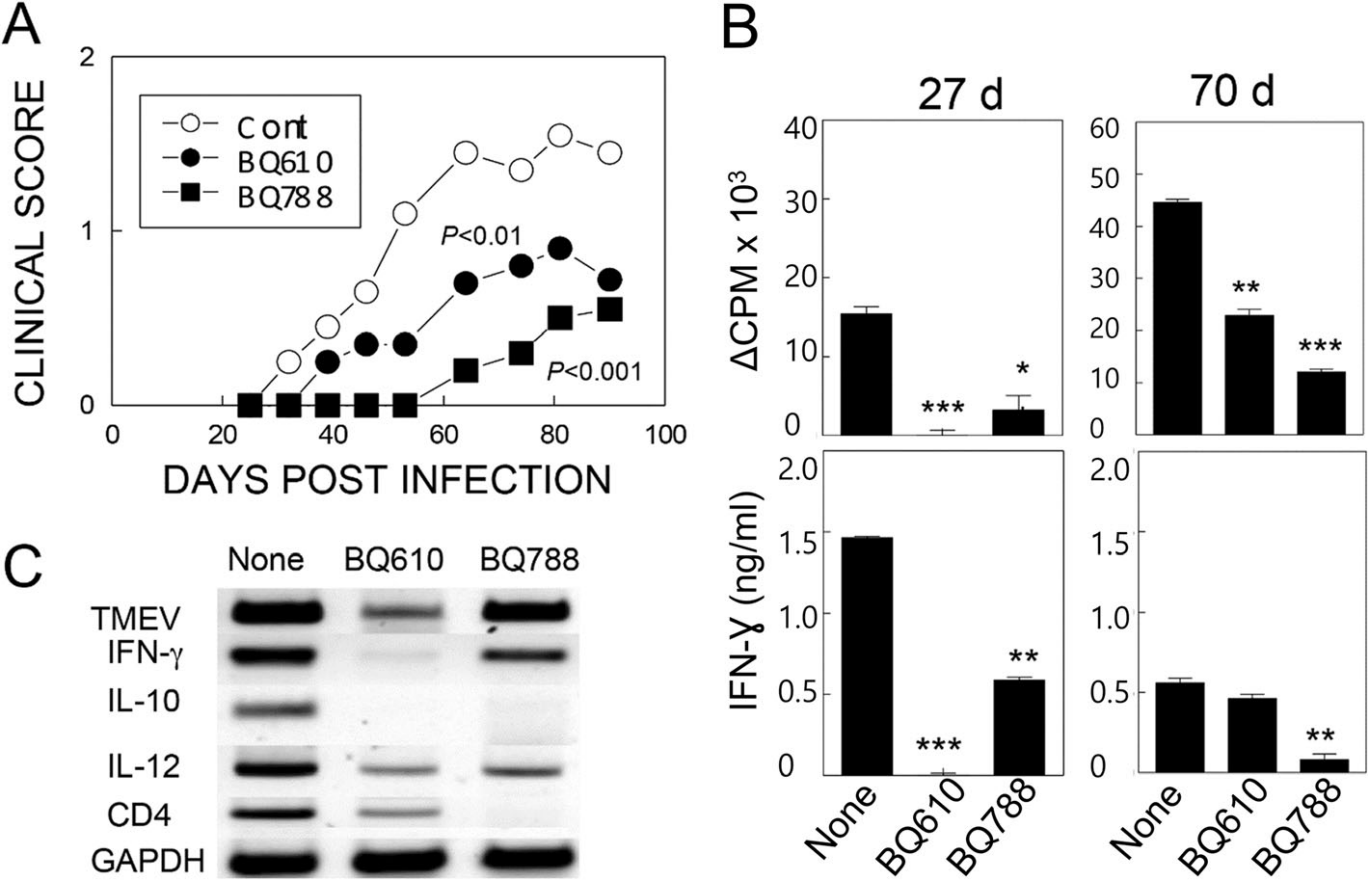
Effects of endothelin receptor antagonists on the development of TMEV-induced demyelinating disease. Mice (*n* = 5/group) were infected with TMEV (1 × 10^5^ PFU) and were treated with PBS (cont), BQ610, or BQ788 (1 mg/kg) at days 0, 5, 10, 15, 20, 30, and 46. **a** Disease course was determined using the 5-point scale. Statistical significance was determined by two-tailed paired *t* test. **b** Proliferative responses and IFN-γ production by splenic T cells to UV-TMEV at 27 and 70 dpi. **c** Comparison of cytokine message levels in the CNS of treated mice at 27 dpi following RT-PCR. Data are presented as mean ± SEM of three independent experiments. *p < 0.05, **p < 0.001, and ***p < 0.0001

To further verify effects of ET-1 on the development of TMEV-induced demyelinating disease, we examined virus-induced demyelination upon administration of SJL mice with ET-1 receptor inhibitors (BQ610 or BQ788) during viral infection. Administration of either ET-1 receptor inhibitor significantly inhibited development of TMEV-IDD (*p* < 0.01) compared to the control group without any inhibitors (Fig. 5a). The treatment with ETB inhibitor (BQ788) (*p* < 0.001) appears to provide better protection from TMEV-IDD compared to ETA (BQ610) treatment (*p* < 0.01), suggesting that endothelin receptor-B plays the major role in the pathogenesis of TMEV-IDD. Levels of T cell proliferative responses and IFN-γ production of splenic T cells against UV-TMEV at 27 and 70 dpi were also examined (Fig. 5b). Proliferation responses were significantly lower to UV-inactivated TMEV in mice treated with ET-1 inhibitors. Similarly, IFN-γ production of the spleen cells in response to TMEV was lower in these treated mice. In addition, mRNA levels of the TMEV virus, IFN-γ, IL-10, and IL-12 cytokines, as well as of the CD4 T cell marker, were visibly lower in the CNS of mice treated with ET-1 receptor inhibitors at 27 dpi (Fig. 5c). These results strongly suggest that the administration of ET-1 receptor inhibitors suppresses the development of TMEV-induced demyelinating disease in susceptible SJL mice.

### Administration of ET-1 accelerated the development of PLP-induced EAE

**Fig. 6:**
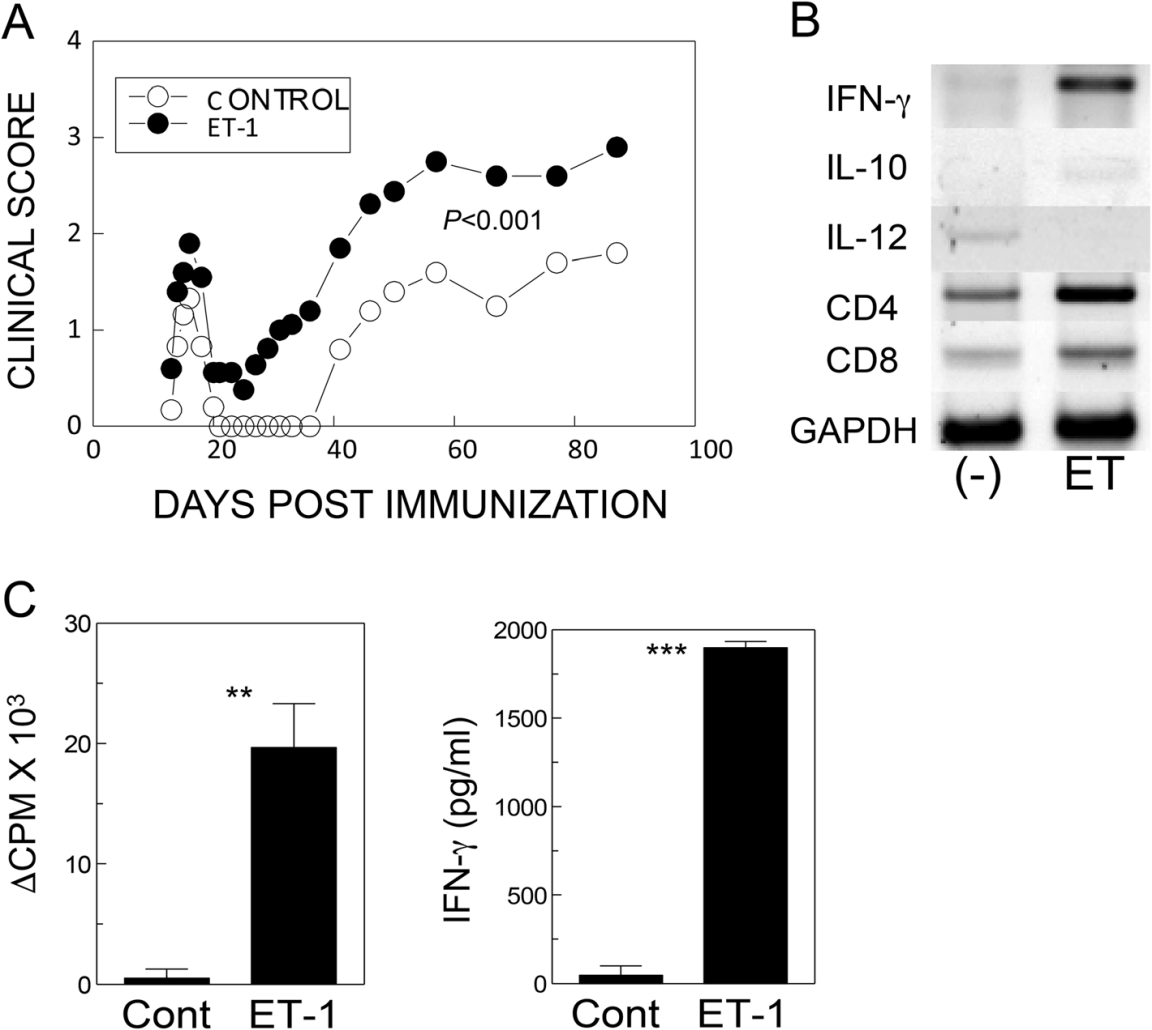
Effect of endothelin-1 on PLP-induced EAE. **a** SJL/J mice (*n* = 5/group) were immunized with a suboptimal dose (10 μg) of PLP and were injected with PBS or endothelin-1 at 20 μg/kg at −3, 0, 5, 10, 15, 20, and 46 dpi. *p* < 0.001 between control and ET-1 treated group based on two-tailed paired *t* test between days 31 and 87 post-immunization. **b** Levels of CD4, CD8, and cytokine mRNA in the CNS of treated mice. **c** Proliferative responses and IFN-γ production by splenic T cells from control and ET-1 treated mice (*n* = 3) to PLP (10 μM) at 20 days after PLP-immunization. Data are presented as mean ± SEM of three independent experiments. **p < 0.001, and ***p < 0.0001

Previously, it was shown that EAE development in transgenic mice overexpressing ET-1 with MOG_35-55_ is exacerbated [30]. To further compare the effect of ET-1 administration on the development of TMEV-IDD with the development of autoimmune-mediated demyelinating disease, SJL/J mice were immunized with the PLP_139-151_ peptide and were treated with either PBS or ET-1 (Fig. 6). The level of EAE development in SJL mice that received ET-1 was significantly (*p* < 0.001) elevated compared to control mice treated with PBS (Fig. 6a). Levels of CD4, CD8, and inflammatory cytokine (IFN-γ, IL-10, and IL12) mRNA in the CNS of ET-1 treated mice were also higher compared to the control mice (Fig. 6b). In addition, proliferative responses (*p* < 0.01) and IFN-γ production (*p* <0.001) by splenic T cells from the ET-1-treated mice to PLP (10 μM) at 20 days post-immunization were also greater than those of control (Fig. 6c). Further experiments showed that EAE development was similarly inhibited after administration of BQ788 (*p* < 0.05), but not BQ610 (Additional file 1: Supplementary Figure 2). These results are consistent with the previous observation with EAE [30] and similar to the effects on TMEV-IDD (Figs. 1 and 5).

## Discussion

Infection of SJL mice with TMEV activates various cellular proteins via TLR2 and TLR3 [14, 15, 34]. ET-1 production is induced via TLR2, TLR3, and TLR4 [31–33]. In addition, ET-1 is produced in many different cell types and is associated with NF-κB activation [35, 36], which plays a critical role in TMEV replication [45, 46]. ET-1 also induces COX-2 expression and PGE2 production [38], which significantly affect the pathogenesis of TMEV-induced demyelinating disease [19]. Therefore, it is plausible that TMEV infection induces ET-1 via TLR signaling and consequently affects development of TMEV-induced demyelinating disease. In this study, we explored the potential role of ET-1 in the development of TMEV-induced demyelinating disease.

First, we compared ET-1 levels in the CNS of SJL mice infected with TMEV and in uninfected control mice at 8 dpi (Fig. 1a). The level of ET-1 mRNA in the CNS was significantly elevated in TMEV-infected mice compared to uninfected mice, suggesting that infection of SJL mice with TMEV results in significantly higher ET-1 levels. This result is consistent with previous observations that viral infection elevates ET-1 production [32, 33]. To further investigate the possible effects of elevated ET-1 on the development of TMEV-induced demyelinating disease, we administered the ET-1 peptide during early viral infection (Fig. 1b). Development of clinical symptoms was highly accelerated (*p* < 0.0024), accompanying elevation of CCL2 and CXCL1 chemokines which affect cellular infiltration in mice treated with ET-1 compared to the control mice. It was previously shown that ET-1 increases production of CCL2, CXCL1, and CXCL8 [47, 48]. Furthermore, these chemokines may also induce ET-1 production [49], which activates an amplification loop for the chemokines and ET-1. ET-1 is also known to enhance VCAM-1 expression on vascular endothelial cells and the dysfunction of endothelial cells [37]. This overproduction of chemokines, cytokines, and ET-1 in TMEV-infected mice may contribute to the pathogenesis of viral inflammatory disease by promoting cellular migration into the CNS. We also confirmed that the presence of a high level of ET-1 significantly elevates development of PLP_139-151_-induced EAE in SJL mice (Fig. 6a), as shown previously in MOG_35-55_-induced EAE in C57BL/6 mice [30].

To verify the cellular migration into the CNS, cell types that infiltrated the CNS after TMEV infection were compared between the groups treated with ET-1 and PBS (Fig. 2). ET-1-treated mice showed significantly higher proportions of DCs, NK cells, macrophages, and granulocytes in the CNS compared to the control group during early viral infection. Levels of both IFN-γ and IL-17 mRNAs were significantly higher in CD4^+^ T cells, and only the IFN-γ mRNA level was higher in CD8^+^ T cells in ET-1-treated mice (Fig. 3). Histological examination of the spinal cords and brains of mice infected with TMEV at 30 dpi showed that there are more infiltrating lymphocytes/macrophages that accumulated in both the spinal cord and cerebellum of ET-1-treated SJL mice, but these accumulated only in the spinal cord of the control SJL mice (Fig. 4). Cellular infiltration was mainly present in the white matter in control SJL mice in contrast to the infiltration into the gray matter of the spinal cord and cerebellum of the ET-1-treated SJL mice. It was previously shown that development of TMEV-induced demyelinating disease accompanies cellular infiltration that is limited to the white matter in the spinal cord [8, 50], as seen in the TMEV-infected control SJL mice. However, ET-1 treated TMEV-infected SJL mice showed that the cellular infiltration extended to the gray matter of the spinal cord and brain. These results strongly suggest that excess levels of ET-1 during viral infection further exacerbates cellular infiltration and inflammation in the CNS.

However, the above experiments do not directly prove the direct involvement of ET-1 in the pathogenesis of TMEV-induced demyelinating disease. As such, we further investigated the potential role of ET-1 in the development of TMEV-induced demyelinating disease by administering ET-1 receptor inhibitors (BQ610 or BQ788) during viral infection (Fig. 5). Administration of either ET-1 receptor inhibitor significantly decreased development of TMEV-IDD accompanied with decreased levels of T cell responses to the virus. ET-1 receptor inhibitors have previously been successfully used to define the role of ET-1 in various clinical and experimental diseases [51–53] including EAE [29]. Thus, the lack of ET-1 function during viral infection inhibits TMEV-IDD development, indicating that the presence of ET-1 during viral infection significantly contributes to the pathogenesis of TMEV-induced demyelinating disease in susceptible SJL mice.

## Conclusions

Thus, we concluded that the presence of ET-1 plays an important role in the pathogenesis of TMEV-induced demyelinating disease. TMEV infection triggers ET-1 production, most likely via TLRs [14, 15, 31–34]. Consequently, TLR-mediated signals activate the NLRP3 and NF-κB pathways, which are associated with production of various inflammatory chemokines and cytokines, as well as with viral replication [19, 21, 45, 46]. These chemokines and cytokines may be further engaged in an amplification loop of ET-1, which are involved in cellular infiltration into the CNS and in promoting inflammatory responses [47–49]. In addition, ET-1 and NLRP3 that are activated following TMEV infection [19] may also participate in PGE2 production [38], causing a skew to the development of pathogenic Th17 and Tc17 responses [5, 6].

## Supplementary information

**Additional file 1: **Supplementary Figure 1 Effects of endothelin administration on the expression of inhibitory and co-stimulatory molecules on monocytes (CD11b^+^) in the CNS of infected mice. The percentage of inhibitory (PDL-1) and co-stimulatory molecules (CD40, CD80, CD86, and MHC) expressed on CNS CD11b^+^ cells among the total CNS CD11b^+^ cells from TMEV-infected mice treated with either PBS or ET-1 (each, n = 3) at 8 dpi (mean ± SD). Data are representative of three independent experiments. *, *p* < 0.05, and **, *p* < 0.001. Supplementary Figure 2. Effect of endothelin receptor antagonist treatment on PLP-induced EAE. Mice (n=5/group) was immunized with optimal dose (40 μg PLP) and treated with PBS (cont), BQ610, or BQ788 (1 mg/kg) at 0, 5, 10, 15, 20, 30, and 46 dpi. The disease course was determined using the 5-point scale. BQ788 treated group was significantly (p<0.045) different from other groups based on two-tailed paired t test between 50-76 dpi.

## Data Availability

The datasets supporting the conclusions of this article are included within the article and its additional files.

## Abbreviations

MS: Multiple sclerosis
TMEV: Theiler’s murine encephalomyelitis virus
TMEV-IDD: TMEV-induced demyelinating disease
EAE: Experimental allergic encephalitis
CNS: Central nervous system
