# Supplementary material for: Endothelin-1 contributes to the development of virus-induced demyelinating disease
Source: J Neuroinflammation. 2020 Oct 17;17:307. doi: 10.1186/s12974-020-01986-z (PMC7568825; doi:10.1186/s12974-020-01986-z)

Supplementary Figure 1

Effects of endothelin administration on the expression of inhibitory and co-stimulatory molecules on monocytes (CD11b+) in the CNS of infected mice. Percentage of the inhibitory (PDL-1) and co-stimulatory molecules (CD40, CD80, CD86, and MHC) expressed CNS CD11b^{+} cells among total the CNS CD11b^{+} cells in TMEV-infected mice treated with either PBS or ET-1 (each, n = 3) at 8 dpi (mean ± SD). Data are representative of three independent experiments. *, $p < 0.05$, and **, $p < 0.001$.

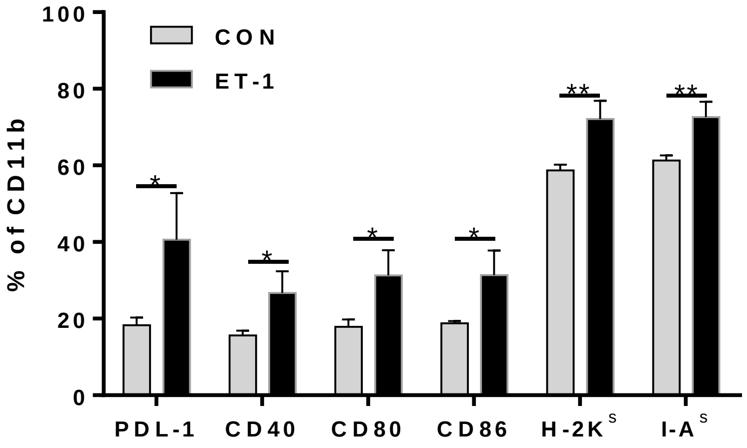

Supplementary Figure 2

Effect of endothelin receptor antagonist treatment on PLP-induced EAE. Mice (n=5/group) was immunized with optimal dose (40 μg PLP) and treated with PBS (cont), BQ610, or BQ788 (1 mg/kg) at 0, 5, 10, 15, 20, 30, and 46 dpi. The disease course was determined using the 5-point scale. BQ788 treated group was significantly (p<0.045) different from other groups based on two-tailed paired t test between 50-76 dpi.

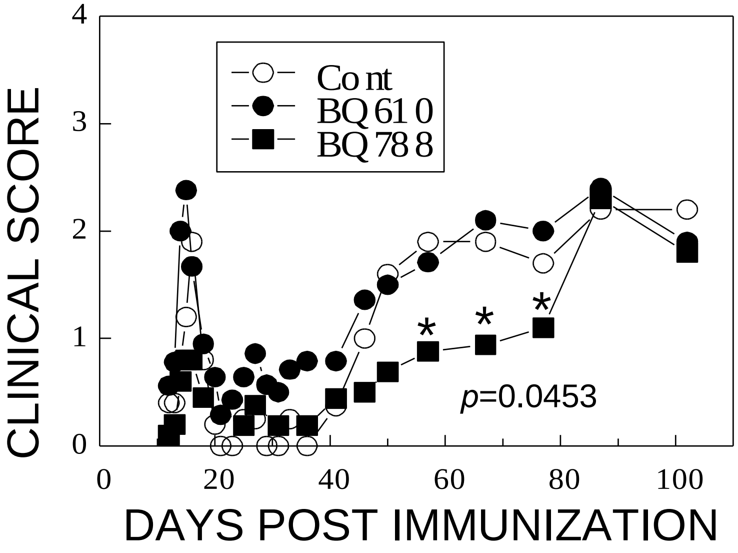

Supplement: Supplementary file 1 — Additional file 1: Supplementary Figure 1 Effects of endothelin administration on the expression of inhibitory and co-stimulatory molecules on monocytes (CD11b+) in the CNS of infected mice. The percentage of inhibitory (PDL-1) and co-stimulatory molecules (CD40, CD80, CD86, and MHC) expressed on CNS CD11b+ cells among the total CNS CD11b+ cells from TMEV-infected mice treated with either PBS or ET-1 (each, n = 3) at 8 dpi (mean ± SD). Data are representative of three independent experiments. *, p < 0.05, and **, p < 0.001. Supplementary Figure 2. Effect of endothelin receptor antagonist treatment on PLP-induced EAE. Mice (n=5/group) was immunized with optimal dose (40 μg PLP) and treated with PBS (cont), BQ610, or BQ788 (1 mg/kg) at 0, 5, 10, 15, 20, 30, and 46 dpi. The disease course was determined using the 5-point scale. BQ788 treated group was significantly (p<0.045) different from other groups based on two-tailed paired t test between 50-76 dpi. [file 12974_2020_1986_MOESM1_ESM.pdf]
